# Sleep and behavioral problems in preschool-age children with Down syndrome

**DOI:** 10.3389/fpsyg.2022.943516

**Published:** 2022-07-18

**Authors:** Elisa Fucà, Floriana Costanzo, Luciana Ursumando, Laura Celestini, Vittorio Scoppola, Silvia Mancini, Diletta Valentini, Alberto Villani, Stefano Vicari

**Affiliations:** ^1^Child and Adolescent Neuropsychiatry Unit, Department of Neuroscience, Bambino Gesù Children’s Hospital, IRCCS, Rome, Italy; ^2^Pediatric Unit, Department of Pediatric Emergency (DEA), Bambino Gesù Children's Hospital, IRCCS, Rome, Italy; ^3^The School of Pediatrics, Tor Vergata University, Rome, Italy; ^4^Department of Human Sciences, European University of Rome, Rome, Italy; ^5^Department of Life Science and Public Health, Catholic University of the Sacred Heart, Rome, Italy

**Keywords:** trisomy 21 (Down syndrome), behavior, child behavior checklist, sleep disturbance scale for children, preschoolers

## Abstract

Sleep is a major concern, especially in people with Down Syndrome (DS). Beyond Obstructive Sleep Apnea, a number of other sleep difficulties have been reported in children with DS, such as delayed sleep onset, night-time awakenings, and early morning awakenings. The detrimental effect of sleep difficulties seems to contribute to and exacerbate the cognitive and behavioral outcomes of DS. Although the screening for sleep disorders is recommended early in age in DS, only a few studies have evaluated the sleep profile in preschool-age children with DS. The aim of the current study was to assess the association between sleep disturbances and behavioral problems in a group of preschool-age children with DS, by means of a feasible and easy-to-administer parent-report questionnaires. Seventy-one preschool-age children with DS, ranging in age from 3 to 5.11 years, were included in this retrospective study. Sleep disturbances were evaluated by means of the Sleep Disturbance Scale for Children, while emotional and behavioral problems by means of the Child Behavior Checklist. Sleep breathing disorders were the most frequent sleep difficulties reported by parents. Moreover, children with clinical scores in total sleep problems exhibited elevation of psychopathological symptoms, namely Total problems, Affective problems, Anxiety problems, Pervasive Developmental Problems, and Attention Deficit/Hyperactivity Problems. The identification of the broader connection between sleep difficulties and emotional and behavioral problems in preschool-age children with DS leads to important considerations for intervention.

## Introduction

Sleep is an active, dynamic neurophysiological function that is ubiquitous across species. Sleep plays a crucial role in neurocognitive and behavioral development, cellular and tissue renewal, as well as in the maintenance of optimal cardiovascular and metabolic functions (Graven and Browne, 2008; Mukherjee et al., 2015). Therefore, it is essential for optimal health and overall quality of life throughout the entire lifespan. Sleep architecture, duration, and quality evolve over a lifetime, especially in the first 5 years of life. For instance, sleep duration fluctuates broadly from infancy to adolescence, with ranges that gradually decrease with age, passing from sleep duration requirements of 12–16 h/day at 4–12 months of age to 8–10 h/day at 13–18 years of age (Paruthi et al., 2016; Carter and Wrede, 2017).

The incidence of parent-reported sleep disturbances in preschool age has been estimated at 10–25% (Goodlin-Jones et al., 2008; Van Litsenburg et al., 2010; Byars et al., 2012; Murthy et al., 2015). Common sleep problems in preschool-age include difficulties falling asleep and night wakings (Ophoff et al., 2018), primary insomnia (Steinsbekk et al., 2013), primary nocturnal enuresis (Wen et al., 2006), frequent nightmares (Simard et al., 2008), primary hypersomnia, and sleepwalking disorder (Steinsbekk et al., 2013). Moreover, some forms of sleep difficulties, such as night waking, are more frequent in preschoolers than in school-age children (Petit et al., 2007; Ravikiran et al., 2011). Preschool children are especially prone to the effects of inadequate sleep and poor sleep quality. The lack of adequate sleep in infancy and childhood has been associated with academic and language difficulties and behavior problems, such as hyperactivity, inattention, impulsivity, anxiety, and depression (Gregory et al., 2005; Touchette et al., 2007; Astill et al., 2012; Armstrong et al., 2014; Shanahan et al., 2014). A number of longitudinal studies identified sleep duration and night waking in preschool age as important predictors of inattentiveness and hyperactive symptoms in childhood (Reynaud et al., 2018, 2021; Huhdanpää et al., 2019; Lewien et al., 2021).

These detrimental effects seem to contribute to and exacerbate the outcomes of neurodevelopmental disorders, in which the prevalence of sleep disorders is higher than typically developing population (TD), with an estimate reported to be as high as 80% (Cotton and Richdale, 2006; Heussler and Hiscock, 2018).

Sleep is a major concern, especially in people with Down Syndrome (DS). The importance of sleep in children with DS has been highlighted by the American Academy of Pediatrics, as the Committee on Genetics published the Health Supervision for Children with DS. These guidelines recommend that all children with DS undergo an overnight diagnostic polysomnography between ages 3 and 4 years (Bull et al., 2022). Children with DS exhibit high rates of Obstructive Sleep Apnea (OSA; 69–76% of estimated prevalence); the prevalence of moderate-to-severe OSA is higher in younger age (Lee et al., 2018). Beyond OSA, a number of other sleep difficulties have been reported in children with DS, such as delayed sleep onset, night-time awakenings, and early morning awakenings, affecting 52–69% of children with DS (Carter et al., 2009; Rosen et al., 2011; Churchill et al., 2015). Sleep problems in children with DS have been associated with a range of behavioral problems, mainly externalizing symptoms (Anand et al., 2021; Fucà et al., 2021).

However, most of the studies exploring sleep difficulties in the DS population most often focused on school-age children (Chawla et al., 2020) or employed cross-sectional approaches including both preschool- and school-age children. Few studies specifically focused on sleep problems in preschool-age children with DS. Among these, Heubi et al. (2021) showed that 30% of children with DS (years 3–5) have ≥2 metrics of poor sleep architecture as measured by polysomnography. Preschoolers with DS show increased sleep breathing disorders (SBD), as well as longer total and daytime sleep duration than TD (Joyce and Dimitriou, 2017). Increasing severity of OSA has been linked to more behavioral abnormalities, including both internalizing and externalizing symptoms (Anand et al., 2021). Conversely, Joyce and Dimitriou (2017) did not report associations between sleep characteristics and behavior in preschool-age children with DS. In addition, studies focusing on cognitive correlates of sleep difficulties in preschool-age reported inconsistent results. For instance, contrasting findings emerge with respect to the association between sleep difficulties and language abilities, with some authors reporting associations between sleep quality and oral language production in preschool children with DS (Edgin et al., 2015), whereas other studies failed to detect this association (Joyce and Dimitriou, 2017).

Since early development is predictive of later developmental outcomes, and considering the important role of sleep function in neurocognitive and behavioral development, exploring the behavioral correlates of sleep difficulties in preschool-age children with DS is essential to provide important indications for prompt, proper interventions. The aim of the current study was to assess the association between sleep disturbances and behavioral problems by means of a feasible and easy-to-administer parent-report questionnaire, in a group of preschool-age children with DS. We hypothesized that: (i) SBD would be the most frequent sleep difficulty reported from parents in our sample and (ii) children with DS with sleep difficulties would have more behavioral problems, in particular, more externalizing problems, than children without sleep difficulties.

## Materials and methods

### Sample size estimation and participant recruitment

The sample size was calculated by *a priori* analysis in G* Power, version 3.1.9.7 (The G*Power Team, Düsseldorf, Germany). We calculated the expected effect size (f) to low and estimated it at 0.35. With an estimated *f* = 0.35, α value = 0.05 (i.e., probability of false positives of 5%), and *β* = 0.80 (i.e., at least 80% power), the sample size that was required for ANOVA with two groups was 68. Seventy-one preschool-age children with DS ranging in age from 3 to 5.11 years of age (mean 4.33 ± 0.81 years; 44 males and 27 females) were included in the study. Selection criteria included, besides the diagnosis of DS based on the analysis of the karyotype, the age ranging between 3 and 5 years. Exclusion criteria were as follows: the ascertained presence or the clinical suspect of neurological conditions, such as West syndrome and epilepsy, language barrier hampering questionnaire compilation by caregivers.

### Procedure

This is a retrospective, cross-sectional study. Data were collected from a file review of preschool-aged children with DS referred for a clinical evaluation at the Pediatric Unit and/or the Child and Adolescent Neuropsychiatry Unit of a pediatric Hospital between June and November 2021. Preschool children with DS underwent a pediatric and/or a neuropsychological evaluation; as part of the clinic visit, caregivers typically complete parent-report measures to investigate the presence of sleep difficulties and psychopathological questionnaires regarding their child. The study was conducted according to the guidelines of the Declaration of Helsinki. All data were de-identified and patients’ confidentiality was protected.

### Measures

Sleep disturbances were assessed by means of Sleep Disturbance Scale for Children (SDSC; Bruni et al., 1996), a questionnaire that has demonstrated through validation an adequate level of internal consistency, test–retest re-liability, and availability of normative data. The SDSC explores the presence of sleep disorders during the previous 6 months and contains 26 items with Likert scale values of 1–5. It is considered “pathological” a T-score > 70 and “suspect/borderline” a T-score between 61 and 70. Due to a different prevalence of sleep disturbances in younger children, for the age range considered in the current study (3–6 years old), it has been proposed a different factorial structure from the original SDSC (Romeo et al., 2013). The most common areas of sleep disturbance in preschoolers were divided into six factors: difficulty in initiating and maintaining sleep (DIMS), sleep-disordered breathing (SDB), parasomnias (PAR), disorders of excessive somnolence (DOES), non-restorative sleep (NRS), and sleep hyperhidrosis (SHY; Romeo et al., 2013).

Emotional and behavioral problems were evaluated by means of the Child Behavior Checklist (CBCL, Achenbach and Rescorla, 2000). We used the CBCL for ages 1.5–5 years, which consists of 100 problem items. There are seven syndrome scales: Emotionally Reactive, Anxious/Depressed, Somatic Complaints, Withdrawn, Sleep Problems, Attention Problems, and Aggressive Behavior. The summary profile contains the Internalizing, Externalizing, and Total Problems scales. Finally, there are five DSM-oriented scale profiles, consistent with the diagnostic categories of DSM-IV-TR and DSM-5 (Affective problems, Anxiety problems, Pervasive Developmental Problems — PDP, Attention Deficit/Hyperactivity Problems — ADHD, and Oppositional Defiant Problems). For the current study, we considered the summary profile and the DSM-oriented scale profiles.

### Data analysis

Descriptive statistics were used to analyze the demographic characteristics of the whole sample. Correlation analyses were used to investigate possible association between sleep problems and socio-demographic variables (i.e., age and sex). ANOVA was computed on CBCL scores to investigate differences according to the presence/absence of sleep problems. Unequal correction was used as *post-hoc* test. Statistical tests were used with a significance level of *p* < 0.05.

## Results

### Distribution of sleep disturbances and emotional and behavioral problems

The distribution of sleep disturbances in our sample, assessed by the SDSC questionnaire, is summarized in Table 1. SBD was the most frequent sleep problem, with 15.4% of clinical scores registered, followed by DIMS (12.7% clinical scores), and PAR (9.9% clinical scores). The 7.1% of total scores at SDSC were in the clinical range, the 18.3% in the borderline range, whereas the 74.6% of participants registered scores in the non-clinical range.

**Table 1:** Distribution of sleep disturbances in preschool-aged children with DS.

	**DIMS**	**SBD**	**NRS**	**PAR**	**DOES**	**SHY**	**TOT**
Non-clinical scores (%)	77.4	76.1	15.4	81.6	95.8	90.1	74.6
Borderline score (%)	9.9	8.5	8.5	8.5	2.8	8.5	18.3
Clinical score (%)	12.7	15.4	7	9.9	1.4	1.4	7.1

Percentage of non-clinical scores, borderline scores, and clinical scores are reported. DIMS, disorders in initiating and maintaining sleep; SBD, sleep breathing disorders; NRS, non-restorative sleep; PAR, parasomnias; DOES, disorders of excessive somnolence; SHY, sleep hyperhidrosis.

The distribution of emotional and behavioral problems in our sample, assessed by the CBCL questionnaire, is summarized in Table 2. The 14.1% of the total sample exhibited scores in the clinical range for internalizing problems, whereas only the 2.8% displayed pathological scores in the Externalizing problems scale. Finally, the 12.7% of our sample exhibited scores in the clinical range for the Total Problems subscale.

**Table 2:** Distribution of emotional and behavioral problems in preschool-aged children with DS (CBCL summary profile scores and CBCL DSM-oriented scale scores).

	**Internalizing problems**	**Externalizing problems**	**Total problems**	**Affect.**	**Anx.**	**PDP**	**ADHD**	**ODD**
Non-clinical scores (%)	74.6	83.1	77.4	93	93	67.6	94.4	97.2
Borderline score (%)	11.3	14.1	9.9	1.4	1.5	18.3	4.2	1.4
Clinical score (%)	14.1	2.8	12.7	5.6	5.5	14.1	1.4	1.4

Percentage of non-clinical scores, borderline scores, and clinical scores are reported. Affect, Affective problems; Anx, Anxiety problems; PDP, Pervasive developmental problems; ADHD, attention deficit/hyperactivity problems; ODD, oppositional defiant problems.

### Emotional and behavioral problems: differences between children with and without sleep problems

With the aim to explore the presence of possible association between socio-demographic variables (i.e., age and sex) and sleep problems, we performed correlation analyses (Pearson and Spearman correlation, respectively) that failed to detect a significant association between the Total Sleep Scale of SDSC and age (*r* = −0.054, *p* = 0.652) nor sex (*r* = −0.186, *p* = 0.120).

In order to explore differences in emotional and behavioral profiles between children with and without sleep problems, we first distinguished among children who displayed SDSC Total scores in the clinical/borderline range (*N* = 19) and children who exhibited SDSC Total scores in the normal range (*N* = 52). Then, a repeated-measure ANOVA analysis on CBCL, with Sleep Group (Sleep problems/No sleep problems) as between factor and CBCL summary profile scores as within factor, was performed, showing a significant interaction *F* (2,138) = 3.855, *p* = 0.023, $\eta_{P}^{2}$ = 0.05. *Post-hoc* analyses (Unequal N HSD) revealed that children belonging to the Sleep problems group exhibited significantly higher scores in the scale of the Total problems than children belonging to the No sleep problems group (60.21 ± 7.84 and 49.42 ± 7.66, respectively; *p* < 0.001). Conversely, no differences between groups emerged in the Internalizing and Externalizing scales.

**Figure 1:**
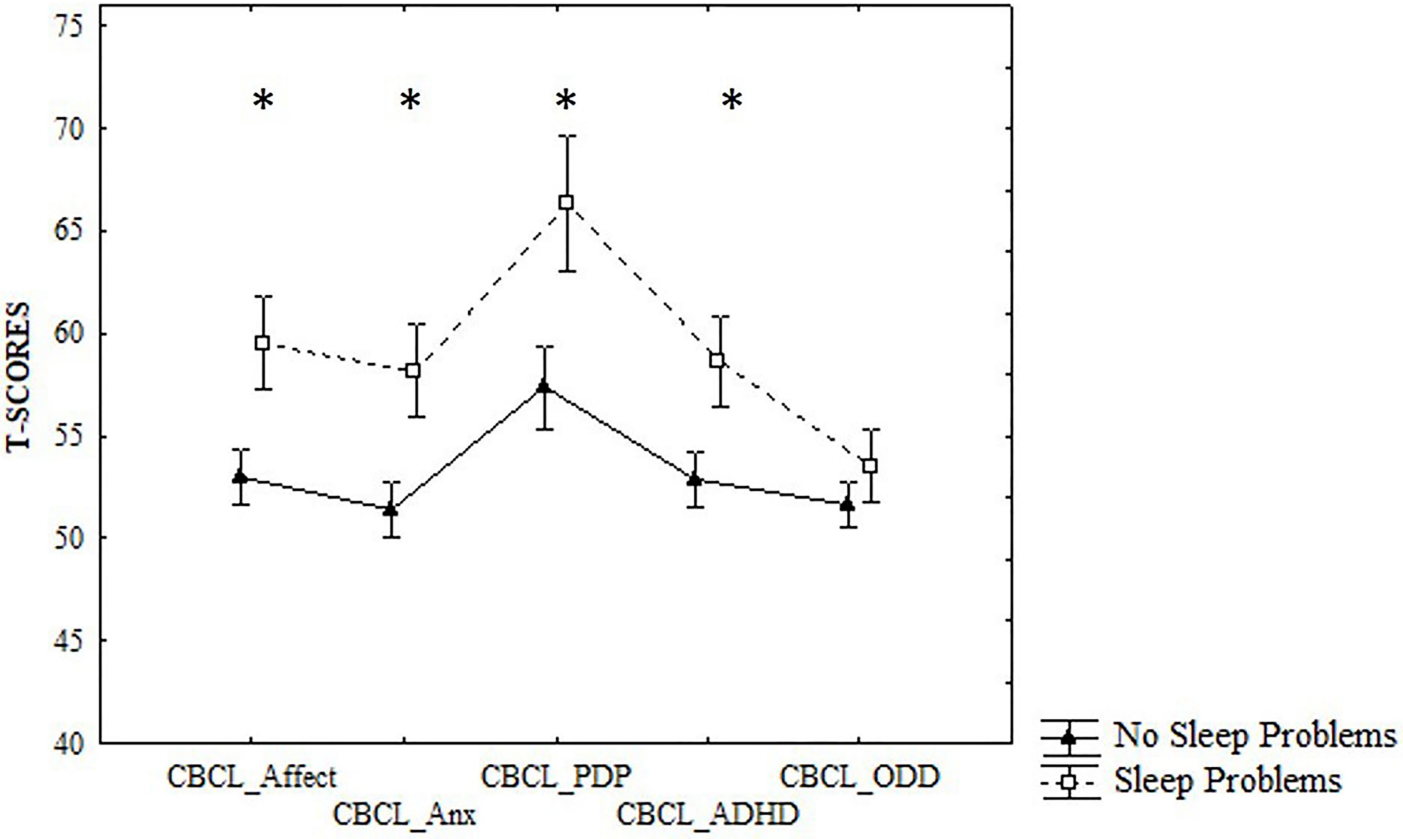
Differences between No Sleep Problems and Sleep Problems groups in CBCL DSM-IV scales scores. *p < 0.05.

Moreover, the ANOVA analysis on CBCL, with Sleep Group (Sleep problems/No sleep problems) as between factor and CBCL DSM-IV scales scores as within factor, was performed, showing a significant interaction *F* (4,276) = 4.54, *p* = 0.001, $\eta_{P}^{2}$ = 0.06 (Figure 1). *Post-hoc* analyses (Unequal N HSD) revealed that children belonging to the Sleep problems group exhibited significantly higher scores than children without sleep problems in the scale of the Affective problems (59.52 ± 7 and 52.94 ± 3.8, respectively; *p* = 0.005), in the Anxiety problems scale (58.16 ± 8.8 and 51.30 ± 2.57, respectively; *p* = 0.003), in the PDP scale (66.32 ± 7.46 and 52.1 ± 7.03, respectively; *p* < 0.001), and in the ADHD scale (58.63 ± 6.88 and 52.86 ± 3.91, respectively; *p* = 0.03), but not in the Oppositional Defiant Problems scale.

## Discussion

In the current study, we retrospectively explored the prevalence of sleep difficulties in a sample of preschool-age children with DS, as measured through a parent-report questionnaire. Consistent with our expectations, SBD was the most frequent sleep difficulty reported by parents, followed by DIMS. We have also investigated the presence of differences in behavioral problems between children with sleep difficulties and children without sleep difficulties. Children with sleep difficulties exhibited significantly higher scores on the Total problems, Affective problems, Anxiety problems, and PDP and ADHD scales of CBCL than children without sleep difficulties.

In spite of the crucial role of sleep in pediatric age, especially for population with DS, very few studies specifically investigated the distribution of sleep difficulties and their behavioral correlates in preschool-age children with DS. On the other hand, the studies that investigated sleep problems in preschoolers with DS included wide age ranges (Levanon et al., 1999; Carter et al., 2009; Nisbet et al., 2014; Bassell et al., 2015; Anand et al., 2021; Chawla et al., 2021; Fucà et al., 2021; Kose et al., 2021). Moreover, studies focusing on preschoolers with DS were conducted on small sample sizes (Edgin et al., 2015; Joyce and Dimitriou, 2017; D’Souza et al., 2020; Lukowski et al., 2020; Arias-Trejo et al., 2021).

To overcome these issues, the current study investigated sleep difficulties in a relatively large group of preschool-age children with DS, focusing on a restricted age range (i.e., 3–5.11 years of age). In our sample, the prevalence of sleep disturbances, as measured *via* SDSC, was lower than that previously reported in studies using parent-report instruments (Yau et al., 2019; Arias-Trejo et al., 2021), although the distribution of sleep disturbances we reported mirrors the literature findings that indicate a higher prevalence of SBD in the DS population. Importantly, the prevalence of sleep disturbances reported in the present study is markedly lower than in those reported in studies that have used objective measures, such as actigraphy or polysomnography, to investigate the presence of sleep problems in preschool-age children with DS. For instance, Edgin and colleagues found that 66% of toddlers with DS included in their study exhibited poor sleep as measured through actigraphy (Edgin et al., 2015); another study found that about 22% of children with DS aged 3 to 5 years have ≥3 metrics of poor sleep architecture as measured by polysomnography (Heubi et al., 2021). Differences in the rate of sleep problems could be due to different reasons, including the different nature of the sample. For instance, Yau et al. (2019) investigated the prevalence of sleep patterns and ecology in 104 children with DS aged 6–36 months, whereas other studies included larger age ranges and did not analyze data from preschoolers separately (e.g., Carter et al., 2009; Rosen et al., 2011; Fucà et al., 2021). Differences between studies in the prevalence rates of sleep problems could be also due to methodological variances, related to the instruments used for the investigation of sleep difficulties in preschoolers with DS. Yau et al. (2019) used the Brief Infant Sleep Questionnaire, whereas Edgin et al. (2015) investigated the presence of sleep difficulties in a group of 29 toddlers with DS by means of an objective measure, i.e., the actigraphy. Similarly, Arias-Trejo et al. (2021) used actigraphy to measure sleep behavior in a group of 36 preschool children with DS. Altogether, these methodological differences could explain, at least in part, the different rates of sleep problems observed in the current study. Of note, the inconsistency between parent-reported sleep difficulties and objective measures highlights the importance of proper psychoeducation for caregivers of children with DS, for instance by the provision of written or verbal information or advice focused on the awareness of sleep hygiene and difficulties. In this sense, psychoeducation should aim to improve caregivers’ understanding of pediatric sleep and prompt identification of potential sleep problems.

The other main finding of the current work was the difference between children with sleep difficulties and children without sleep difficulties in parent-reported behavioral and emotional problems. In particular, it emerged that children with sleep problems, as detected by parents, had significantly higher scores in the Total problems, Affective problems, Anxiety problems, and PDP and ADHD scales of the CBCL. Despite the relatively low prevalence of behavioral problems observed in our sample, this finding suggests that sleep problems in preschoolers with DS should be considered important correlates of behavioral difficulties for this population. Therefore, the detection of sleep difficulties should be considered a “red flag” for possible associated behavioral problems in preschoolers with DS. On the other hand, the presence of behavioral problems should prompt caregivers to pay attention to signs that might suggest the presence of sleep difficulties in their children, e.g., signs of excessive sleepiness during sleep. Given the documented longitudinal effects of sleep disturbance on behavioral problems in children (Gregory et al., 2009; Quach et al., 2009; Astill et al., 2012; El-Sheikh et al., 2013; Armstrong et al., 2014), including children with DS (Horne et al., 2019), the possibility to rely on indicators that allow a prompt recognition should not be underestimated. Moreover, sleep problems also affect daily life abilities and cognitive functions such as attention and language also in children with DS (Horne et al., 2019; Arias-Trejo et al., 2021). Therefore, the proper identification and management of sleep difficulties in preschool-age children with DS could have important consequences on developmental outcomes. The association between poor sleep and behavioral problems has been reported in both TD population and in children with neurodevelopmental disorders. Literature focusing on preschoolers suggests that sleep difficulties are associated with internalizing symptoms in TD children (Lee et al., 2018) and with externalizing symptoms in children with developmental disabilities (Lee et al., 2018; Kang et al., 2020). Studies on the neural bases underlying the possible association between sleep difficulties and behavioral problems in preschoolers are limited. Research on adults reported that the association between sleep quality and the depressive problems was mediated by functional connectivity involving the lateral orbitofrontal cortex, anterior cingulate cortices, hippocampus, and precuneus (Cheng et al., 2018). In adolescence, poor sleep may contribute to depressive affect by disrupting functioning of the dorsal medial prefrontal cortex (Casement et al., 2016). As concerns school-age children, Cheng et al. (2021) reported that depressive problems mediate considerably the effect of low brain area or volume (mostly, the lateral orbitofrontal cortex) on low sleep duration. Future studies are required to explore the mechanisms by which adequate sleep may support behavioral functioning in preschool-age children.

Of note, the most remarkable difference between children with sleep disturbances and children without sleep disturbances emerged for the PDP scale. The PDP scale was constructed based on expert judgment regarding correspondence with Diagnostic and Statistical Manual of Mental Disorders - 4th edition (American Psychiatric Association, 1994) criteria and it includes items such as “Avoids looking others in the eye,” “Disturbed by any change in routine,” “Repeatedly rocks head or body,” and “Withdrawn, does not get involved with others” (Achenbach and Rescorla, 2000). Several studies suggest that the PDP scale of the Child Behavior Checklist for ages 1½–5 years differentiates well between children diagnosed with autism spectrum disorder and those not diagnosed (Rescorla et al., 2015, 2019; Havdahl et al., 2016), also in Italian samples (Muratori et al., 2011). Since the co-occurrence of sleep difficulties in children with autism spectrum disorder has been well-described (Souders et al., 2017; Cortese et al., 2020; Chen et al., 2021), it is possible that this co-occurrence also exists in children with DS and autistic symptoms. Therefore, the prompt identification of sleep problems should be also considered in light of possible neurodevelopmental comorbidities documented in DS, such as autism spectrum disorder (Kirchner and Walton, 2021).

The main limitation of the present study is that the presence of sleep disturbances, detected by a parent-report instrument, was not corroborated by objective measures such as actigraphy or polysomnography. Parent-report questionnaires mainly rely on subjective parental observations; therefore, rater bias cannot be ruled out: for instance, parent-report measures of sleep may underestimate certain measures of child sleep problems. However, previous research suggested that parent-reported sleep problems, but not objective sleep measures, are related to behavioral problems in children with DS (Esbensen et al., 2018). Therefore, the integration of multiple sources of information is highly recommended when studying sleep problems in developmental age, recognizing the specific information each source can provide in order to reach a more comprehensive understanding of the phenomenon (Esbensen et al., 2018). We cannot exclude that shared method variance could have overestimated the observed associations. Another limitation of the study is the descriptive nature of the research, which did not examine potential mechanisms underlying sleep problems nor possible causal associations linking sleep difficulties and behavioral problems in our sample. Finally, the lack of a control group is another limitation of the current study. Future research on the prevalence and behavioral correlates of sleep disturbances in preschoolers with DS should include comparisons with the TD population and other neurodevelopmental disorders.

Nevertheless, the current study has significant clinical implications. Indeed, the identification of the broader connection between sleep difficulties and emotional and behavioral problems leads to an important consideration for intervention planning and targets for preschool-age children with DS. Although the current study cannot establish causal links between sleep and behavior, it is possible to hypothesize that treating sleep problems in preschool-age children with DS may lead to ameliorations in behavioral difficulties. In sum, the results of the current study further highlight the significance of adequate sleep habits and early screening and intervention for sleep problems in children with DS. Future research aiming to understand the whole complexity of the link between sleep and behavioral problems in preschool-age children with DS is required in order to develop appropriate intervention strategies and enhance developmental outcomes.

## Data availability statement

The raw data supporting the conclusions of this article will be made available by the authors, without undue reservation.

## Ethics statement

The studies involving human participants were reviewed and approved by Children Hospital Bambino Gesù. Written informed consent to participate in this study was provided by the participants’ legal guardian/next of kin.

## Author contributions

EF, FC, DV, and SV: conceptualization and writing—reviewing and editing. EF and LU: methodology. EF and FC: formal analysis and writing—original draft preparation. EF, LC, and VS: investigation. LU and SM: data curation. AV and SV: supervision and project administration. All authors contributed to the article and approved the submitted version.

## Conflict of interest

The authors declare that the research was conducted in the absence of any commercial or financial relationships that could be construed as a potential conflict of interest.

## Publisher’s note

All claims expressed in this article are solely those of the authors and do not necessarily represent those of their affiliated organizations, or those of the publisher, the editors and the reviewers. Any product that may be evaluated in this article, or claim that may be made by its manufacturer, is not guaranteed or endorsed by the publisher.
